# Exosomes—nano-messengers in tumors: capturing techniques, birth codes, and functional deciphering

**DOI:** 10.3389/fcell.2026.1825151

**Published:** 2026-08-21

**Authors:** Ying-Ying Li, Ya Gao, Yan-Hong Wang, Kai-Fang Zhou, Yang Zhao, Li-Juan Zhao

**Affiliations:** 1 Department of Pharmacy, The Fifth Affiliated Hospital of Zhengzhou University, Zhengzhou University, Zhengzhou, Henan, China; 2 State Key Laboratory of Metabolic Dysregulation & Prevention and Treatment of Esophageal Cancer, Key Laboratory of Advanced Drug Preparation Technologies, Ministry of Education of China, Key Laboratory of Henan Province for Drug Quality and Evaluation, Henan Province, Institute of Drug Discovery and Development, School of Pharmaceutical Sciences, Zhengzhou University, Zhengzhou, Henan, China; 3 State Key Laboratory of Metabolic Dysregulation & Prevention and Treatment of Esophageal Cancer, School of Basic Medical Sciences, Zhengzhou University, Zhengzhou, Henan, China

**Keywords:** biogenesis, exosomes, immunity, isolation and identification, metastasis, secretion

## Abstract

Exosomes are nanoscale, membrane-bound vesicles secreted by various cell types. Owing to their cargo of parental cel-derived cellular components, exosomes show significant promise as clinical biomarkers. Meanwhile, due to their diverse origins and cargo, exosomes play a dual role in cancer, possessing the capacity to both promote and suppress tumor progression The efficient and rapid isolation and purification of exosomes are foundational for advancing their clinical applications. Additionally, a comprehensive understanding of exosome biogenesis is essential to elucidate their underlying biological functions. However, current knowledge regarding their biological characteristics and functions of action remains limited. This review delves into exosome isolation techniques, the regulatory mechanisms underlying their biogenesis and secretion, and their dual roles in cancer progression, metastasis, and tumor immunity, concluding with a discussion of the challenges and future directions in their clinical translation.

## 1 Introduction

In 1983, R.M. Johnstone et al. first discovered that mature reticulocytes from sheep could release small membrane vesicles, which was originally thought to be used to excrete excess transferrin receptors. In 1987, these small vesicles were named “exosomes” (Johnstone et al., 1987), and they belong to a type of extracellular vesicles (EVs). Exosomes are EVs produced by all cells under physiological and pathological conditions. The diameter of exosomes ranges from 40 to 150 nm, and their density is 1.13–1.19 g·mL^-1^ (Gholizadeh et al., 2017). Exosomes are widely distributed in various body fluids, with previous studies reporting their successful isolation from blood (Caby et al., 2005), urine (Pisitkun Et Al., 2004), breast milk (Admyre et al., 2007), cerebrospinal fluid (Vella et al., 2007), saliva (Ogawa et al., 2011), amniotic fluid (Asea et al., 2008), ascites fluid (Andre et al., 2002), bile (Masyuk et al., 2010), semen (Aalberts et al., 2012), bacterial cultures (Kovach et al., 2016), and other biological fluids. What’s more, exosomes carry parental cell-specific components, including lipids, proteins, messenger RNAs (mRNAs), microRNAs (miRNAs), long non-coding RNAs (lncRNAs) and even DNA, which are exported to the extracellular environment via these vesicles. They play an important part in the transfer of materials and information between cells.

**FIGURE 1:**
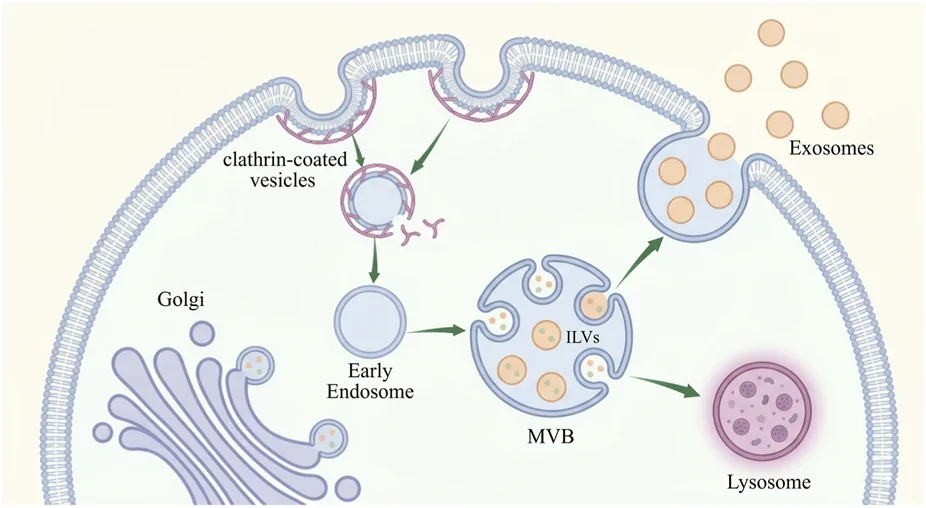
Schematic illustration of exosome biogenesis and secretion. Intracellular proteins and lipids are internalized via clathrin-coated vesicles and fuse with the Early Endosome. The early endosome matures into the Multivesicular Body (MVB), where intraluminal vesicles (ILVs) are formed by inward budding. MVBs can either fuse with lysosomes for degradation or fuse with the plasma membrane to release Exosomes into the extracellular space.

In addition to exosomes, other types of EVs exist. Based on their morphological features and molecular contents, EVs are generally classified into three main categories: exosomes, microvesicles, and apoptotic bodies (Pugholm et al., 2015). Microvesicles are vesicles with a molecular diameter of 100–1,000 nm. They are produced by detaching directly from the surface of the parental cell membrane through budding, and their sizes are not uniform. Apoptotic bodies are formed by the rupture of apoptotic cells, and their diameter is 500–4,000 nm (EL Andaloussi et al., 2013). Different from the general method of budding, the formation and release process of exosomes is that the parental cell releases particulate matter into intraluminal vesicles (ILVs) formed by the plasma membrane invagination. Subsequently, the ILVs mature into multivesicular bodies (MVBs),then MVBs fuse with plasma membrane of the parental cell to release exosomes to the extracellular environment (Figure 1) (Jia et al., 2017).

**FIGURE 2:**
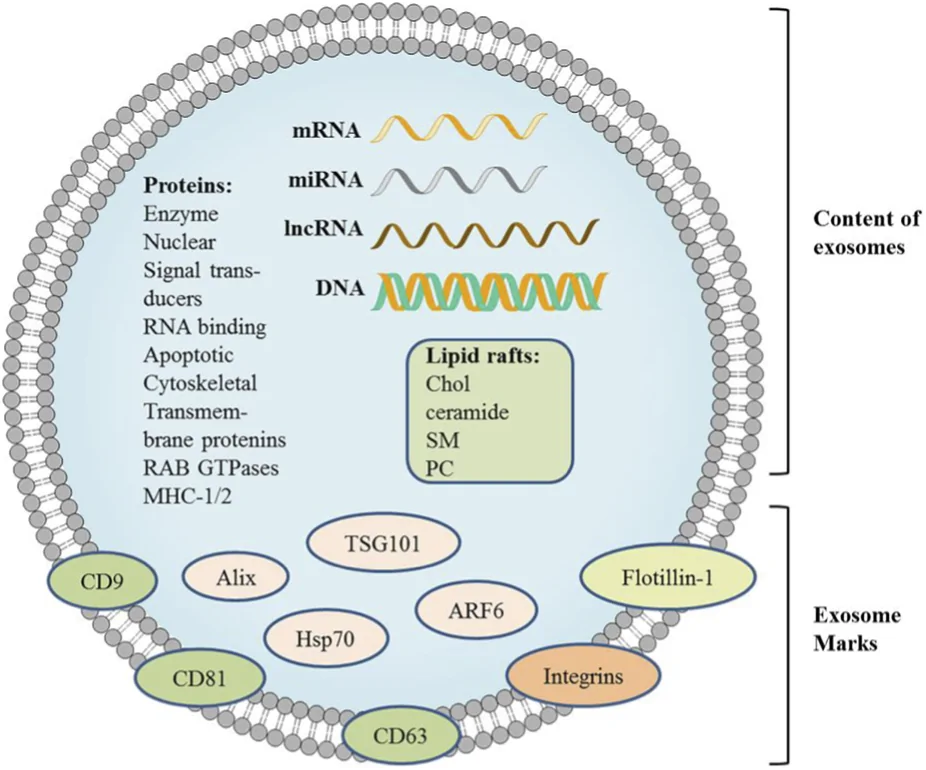
Exosome cargos and markers. The components of exosomes include: A variety of proteins, nucleotide (DNA, RNA) and lipids. The marker proteins of exosomes are Alix, TSG101, tetraspanins (CD9, CD63, and CD81), flotillin-1 and so on.

An increasing number of studies have demonstrated that exosome biogenesis is not merely a mechanism for cells to excrete waste from cells, because they are loaded with functional molecules. The proteins, miRNAs, lncRNAs, circRNAs, mRNAs and their degraded fragments, which are involved in intracellular signal transduction, were encapsulated and protected in the exosomal membrane (Figure 2). Exosomes fuse with recipient cells to release their contents, thereby regulating intracellular signaling and cellular activities. Given that exosomes harbor parent cell-specific proteins and nucleic acids, they can be used for diagnosis, therapeutic efficacy assessment, prognostic monitoring, etc. Based on the advantages of high stability and specificity, an increasing number of studies have confirmed exosomes’ significant potential as biomarkers for tumors and other diseases (Li et al., 2024). Therefore, the isolation and purification of exosomes have garnered considerable attention from scientific researchers, as the rapid and efficient isolation of exosomes is critical for advancing their clinical applications.

Despite this immense clinical promise of exosomes as non-invasive diagnostic biomarkers and targeted drug delivery vectors in oncology, their successful transition from bench to bedside is hindered by substantial translational bottlenecks. As highlighted in recent analyses of global regulatory frameworks, a primary barrier is the complex regulatory classification of exosome-based therapeutics, which often blur the lines between traditional biologics and advanced therapy medicinal products (ATMPs). Consequently, navigating the regulatory approval process necessitates the establishment of robust, scalable manufacturing protocols. Furthermore, rigorous quality control (QC) standards are imperative to address inherent exosome heterogeneity and ensure consistent safety, purity, and potency across batches. The design of subsequent clinical trials must also adapt to these unique biological characteristics, demanding standardized characterization to yield reproducible clinical outcomes (Verma and Arora, 2025). Therefore, this review aims to systematically outline the key characteristics of exosomes at the levels of biogenesis, isolation, functionality, and clinical application, thereby providing theoretical foundations and practical references for a smooth transition of exosome-based research from bench to bedside.

Literature Search and Review Design: This is a narrative review aimed to comprehensively summarize the basic biology, tumor-related roles and clinical translation of exosomes. The literature search was conducted in two major databases: PubMed and Web of Science, using the core keywords including “exosome, exosome isolation, exosome biogenesis, cancer progression, tumor immunity, clinical translation”, covering English original studies, reviews and guidelines published from from January 1987 to March 2025, with the addition of classic landmark studies in the field. Inclusion criteria: high-quality original studies, authoritative reviews and expert consensus related to exosome isolation techniques, biogenesis regulation, tumor-related roles, and clinical translation; Exclusion criteria: duplicate publications, low-quality case reports, and non-English literature.

## 2 Exosome isolation

The development of efficient, rapid, and stable methods to isolate the exosomes is the basis and prerequisite for the current application of exosomes in clinic. Numerous methods for exosome extraction from cell supernatants and body fluids have been reported, and the purity and yield of exosomes are closely related to the method of separation.

### 2.1 Differential centrifugation

Differential centrifugation is widely used to separate EVs subpopulations based on size and density, and is recognized as the “gold standard” and most commonly employed technique for exosome isolation. As documented in published protocols, centrifugal forces of 10,000–20,000 × g are typically employed to deplete relatively large and dense EVs such as microvesicles, whereas forces of 100,000–200,000×g are applied to pellet smaller less dense EVs such as exosomes (Welsh et al., 2024). Based on these principles, we utilized a sequential differential centrifugation method to isolate exosomes from cell culture supernatants or biological fluids. Briefly, samples were subjected to low-speed centrifugation at 400 *g*, 2000×g, 10,000×g to systematically remove cells, cell debris and larger microvesicles, respectively. Subsequently, the target exosomes are pelleted via high-speed ultracentrifugation at 100,000×g for 1–2 h, followed by a washing step with phosphate-buffered saline (PBS). High levels of triglycerides or cholesterol esters in isolated exosomes indicate that samples are readily contaminated with lipid droplets or lipoproteins (Skotland et al., 2017). This method is operationally simple, requires no sophisticated technical support, and features low cost, thus being widely adopted.

Nevertheless, differential centrifugation also presents several limitations. First, this approach is time-consuming and yields impure preparations, with poor recovery of the input extracellular vesicles (Lane et al., 2015). Second, the quantity and quality of isolated exosomes are strongly affected by rotor type and rotor angle. More importantly, exosomes purified by differential centrifugation may be mixed with other vesicles, proteins, or protein-RNA aggregates. In addition, high centrifugal force may damage exosome structure and subsequently compromise downstream experiments (Lai et al., 2022). However, this protocol can be optimized based on characteristics of particles in solution and rotor properties to isolate target vesicle populations with higher yield and purity (Livshits et al., 2015).

### 2.2 Density gradient centrifugation

Studies have reported that the buoyant density of exosomes ranges from 1.13 to 1.19 g/mL. The density gradient centrifugation method is a refined ultracentrifugation approach that enables further separation of vesicles with different densities, and thus is widely employed for exosome isolation (Vidal et al., 1997). The gradient medium initially used was sucrose, so this technique is also called sucrose density gradient ultracentrifugation. This method forms a continuous low-to-high density layer in the sucrose solution under ultracentrifugal force via a zone separation mode. The principle of this method is to remove non-vesicular material first, and then to centrifuge the density gradient, the exosomes in the sample will be enriched in the density range of 1.13–1.19 g/mL. The exosomes obtained by this method have high purity, but the steps are complicated and time-consuming (Martins et al., 2023). Traditional sucrose density gradient methods usually require 16 h of centrifugation. If the centrifugation time is not sufficient, the contaminated material and the exosomes may remain in the same density layer, especially as this density range is relatively wide. With continuous methodological optimization, iodixanol has become a high-performance alternative gradient medium. Compared with sucrose, iodixanol displays prominent advantages: as a non-ionic, iso-osmotic and low-viscosity medium, it exerts minimal osmotic damage and biological toxicity to exosomes, better preserves the structural and biophysical properties of vesicles, and more efficiently removes impurities such as large protein aggregates. This optimized medium outperforms conventional ultracentrifugation in maintaining vesicle integrity and eliminating contaminants. For standard iodixanol density gradient centrifugation, typical operating parameters are 100,000–120,000×g for 60–180 min at 4 °C, and fraction collection is recommended to be performed sequentially from the top to the bottom of the centrifuge tube with 100–200 μL per fraction to ensure high separation resolution. Nevertheless, the iodixanol gradient protocol remains constrained by its relatively labor-intensive operation, representing a remaining challenge for large-scale exosome preparation (Wu et al., 2025).

### 2.3 Polymer precipitation

Polymer precipitation was previously used to collect viruses and other biological macromolecules from samples, and is now also used as a method to precipitate exosomes. This method uses high-molecular- weight hydrophobic polymers, such as polyethylene glycol (PEG), which can change the solubility and dispersibility of exosomes, allowing them to settle out under relatively low centrifugal forces. The principle may be related to the competitive binding of free water molecules (Yamada et al., 2012). The main component of the kit for extracting exosomes with PEG solution is PEG8000 (30%–50%). The mixture is incubated with body fluids or cell culture solution at 4 °C overnight, followed by low-speed centrifugation. The advantage of this method is that it is relatively simple to operate, does not require an ultracentrifuge, and yields a greater number of exosomes, which is also suitable for large- volume sample processing (Alvarez et al., 2012). However, exosomes isolated via this method suffer from low purity and recovery efficiency, along with severe heteroproteins protein contamination and persistent polymer residues that are hard to eliminate (Rekker et al., 2014). By reducing the solubility of EVs and soluble proteins, this method inevitably co-precipitates lipoproteins and Ago-2 associated RNA, which would impede the downstream molecular characterization of purified EVs. More importantly, co-precipitation of non-vesicular contaminants (e.g., soluble proteins, lipoproteins and residual PEG polymers) is a prevalent limitation of precipitation-mediated exosome isolation, as these impurities can markedly interfere with critical downstream assays including mass spectrometry, RNA profiling and proteomic analysis. To address this issue and minimize co-purified impurities, a preliminary 0.22 μm filtration step can be implemented, or post-isolation refinement strategies (e.g., additional centrifugation, membrane filtration or size-exclusion chromatography) can be employed (Zhu et al., 2020).

### 2.4 Ultrafiltration

Ultrafiltration is also a method of separating exosomes based on their size. This method is to filter the solvent and small molecular substances to the other side of the membrane, while retaining relatively large molecular substances on the ultrafiltration membrane to achieve the purpose of separation. Given that the diameter of exosomes ranges from 40 to 200 nm, membranes with pore sizes between 0.001 and 0.1 μm are commonly utilized. Typically, ultrafiltration is applied to separate exosomes from samples following prolonged low-speed centrifugation (Cheruvanky et al., 2007). The advantage of this method is that no special equipment is needed, the operation is convenient, the enrichment efficiency is relatively high, and the cost is low. The disadvantage is that the adhesion of the ultrafiltration membrane to the exosomes will reduce the recovery of the exosomes, and the external force applied during the ultrafiltration process may deform or rupture the exosomes. As a result, the membrane is blocked and the purity is low. Ultrafiltration can be performed in two modes: dead-end filtration for small-scale lab use, and tangential flow filtration (TFF), which is preferred for large-scale production due to reduced membrane fouling and higher processing efficiency. Compared with dead-end filtration, TFF operates by passing the sample parallel to the membrane surface, which significantly reduces membrane clogging caused by exosome adhesion and sample accumulation-effectively addressing the membrane clogging issue mentioned later. Moreover, TFF enables continuous large-volume sample processing with higher recovery rates, as it minimizes exosome deformation or rupture caused by external pressure, making it more suitable for large-scale EV enrichment in preclinical and translational studies (Visan et al., 2022).

### 2.5 Immunomagnetic separation

Immunomagnetic separation is a method that exploits the highly specific affinity between the antigens and antibodies for the isolation of targets. It is mainly applied for the separation and purification of biological macromolecules. Exosomes harbor a variety of specific membrane proteins on their exosomes, such as CD9, CD63, CD81, and Annexin. These exosomal marker proteins can serve as specific targets for exosomes isolation. Antibodies (Tauro et al., 2013) and peptide ligands (Da Fonseca Alves et al., 2024; Shao et al., 2020) are immobilized on substrates such as magnetic beads, and mediate specific interactions with these marker proteins to enable the enrichment of exosomes. The advantages of this method include higher exosome purity and minimal disruption to exosomal membrane integrity. Its disadvantages involve high cost, low yield, incompatibility with large-scale extraction, and poor stability of magnetic beads during storage. Liu et al. (2024) employed immunomagnetic separation to isolate exosomes from plasma and cell culture supernatants. The results revealed that multiple marker proteins (Alix, CD9, CD63) were present on the surface of the isolated exosomes. Meanwhile, ElISA and PCR analyses further validated the feasibility of the method. Tauro et al. reported that for exosomes isolation from colon cancer cell lines, the immunoaffinity capture approach yields exosomes of higher purity and exhibits greater efficiency than ultracentrifugation and density gradient methods (Tauro et al., 2006).

### 2.6 Size exclusion chromatography (SEC)

This method mainly uses porous gel spheres to purify exosomes through different molecular sizes, and its principle is similar to the purification of proteins by chromatography (Bing et al., 2014). In the process of separation and extraction, macromolecules cannot enter the gel pores, and are quickly eluted by the mobile phase along the gaps between the beads, while small molecules enter the pores, resulting in a longer retention time and being eluted more slowly. Commonly utilized stationary phases include cross-linked agarose resins, such as Sepharose CL-2B, or standardized commercial platforms like qEV columns. Since the molecular size of exosomes is larger than that of most soluble proteins, exosomes are eluted first, typically within the initial 3.0–5.0 mL elution volume (depending on the column’s void volume), while smaller proteins are eluted later, thereby isolating high-purity exosomes (Nordin et al., 2015). The primary advantage of this approach is that large centrifugal force are not required, thus ensuring the structural and functional integrity of the exosomes. However, a significant disadvantage is the co-elution of particles with similar hydrodynamic radii, particularly triglyceride-rich lipoproteins such as chylomicrons and very low-density lipoproteins (VLDL). While low-density lipoproteins (LDL) are also a concern, the overlap of VLDL (30–80 nm) with small EVs poses a particular challenge for purity. In order to improve the efficiency and resolution of the separation, several factors must be considered, including the type of media, pore size, interactions between EVs and media, column size, column packing, and flow rate.

While SEC effectively separates exosomes from bulk proteins, it may not sufficiently resolve them from microvesicles, protein aggregates, or liposomes. To overcome these limitations, orthogonal separation strategies are increasingly recommended to achieve superior purity. On one hand, coupling SEC with density gradient centrifugation leverages differences in particle buoyant density, effectively separating EVs from dense protein aggregates. On the other hand, immunoaffinity capture serves as a refined orthogonal approach that relies on unique surface markers expressed on vesicle membranes and is typically combined with SEC to enable specific, targeted isolation of EVs (Kim et al., 2025). Notably, Mol et al. reported (Visan et al., 2022) that the protein abundance of exosomes extracted by this method was higher than that by the differential centrifugation method. Overall, by optimizing column parameters and incorporating orthogonal steps, this method provides a viable possibility for the mass production and clinical application of exosomes.

### 2.7 Microfluidics

Conventional exosome isolation methodologies, primarily represented by differential centrifugation and SEC, typically necessitate milliliter-scale input volumes, thereby imposing a significant bottleneck for the analysis of scarce or precious clinical specimens. Furthermore, in these traditional workflows, the separation and detection of samples are usually performed as discrete steps. From the isolation and purification of exosomes to the final morphological and molecular biological identification, there are several procedures. A fast and easy way to integrate separation and detection is essential. Therefore, in recent years, the use of microfluidic technology to separate exosomes has attracted the attention of many scholars. Microfluidics is a technology involved in processing and manipulating fluids using micro-nanoscale pipes, including: acoustic fluid technology (Fong et al., 2014), dielectrophoresis technology (Feng et al., 2007), viscoelastic microfluidics (Liu et al., 2017), flow field-flow fractionation technology (FIFFF) (Kang et al., 2008) and so on. The separation method based on microfluidic technology has the advantages of faster separation rate, higher separation yield and purity, and minimal sample demand (often at the microliter, μl, scale). However, a primary constraint of contemporary microfluidic devices is their relatively low volumetric throughput, with typical flow rates restricted to tens of μl/min. While conventional bulk-processing methods are inherently better suited for handling large-scale volumes (from milliliters to liters), the throughput of microfluidic platforms can be significantly augmented through device parallelization and channel multiplexing (Hassanzadeh-Barforoushi et al., 2025). The field is further advancing toward intelligent, multimodal integration. For instance, Verma et al. developed an AI-driven microfluidic immunoaffinity platform integrated with real-time nanoparticle tracking, enabling the standardized isolation of high-purity EV subsets tailored for ophthalmic applications (Verma et al., 2025a). Ultimately, compared to traditional bulk isolation, microfluidic technology is uniquely positioned to excel in micro-sample analysis. By evolving into comprehensive “sample-to-answer” systems, these platforms hold immense promise for driving future innovations in personalized medicine and precision therapeutics (Zhao et al., 2025).

### 2.8 Dielectrophoresis, DEP

The principle of DEP separation relies on the polarization of particles within a non-uniform electric field, where the resultant force is dictated by the dielectric properties and size of the vesicles, the field frequency, and the electrical conductivity of the suspending medium. While exosomes are typically attracted to high electric field region (positive DEP), their electrokinetic behavior is highly sensitive to the ionic strength of the environment. To maintain a stable dielectrophoretic force and suppress excessive Joule heating—which can lead to thermal degradation of exosomal membranes and cargo—the medium conductivity must typically be optimized to a low range of 0.1–10 mS/m (10–100 μS/cm). Consequently, physiological fluids such as plasma, serum, or undiluted urine, which possess high intrinsic conductivities (∼1,500 mS/m), necessitate rigorous pre-treatment prior to DEP manipulation (Luna et al., 2023). Standard protocols include buffer exchange via dialysis or centrifugal desalting columns, as well as significant dilution into isotonic, low-conductivity buffers (e.g., 8.5% sucrose or 0.3 M dextrose solutions). These steps ensure a favorable Clausius-Mossotti factor for precise exosome capture while preserving the structural and functional integrity of the vesicles. Recently, Stanley et al. designed a unique microfluidic device based on the DEP force and electrohydrodynamic resistance, demonstrating the ability to resolve exosomes from complex biological fluids by carefully balancing conductivity and flow parameters (Hassanpour et al., 2021). By integrating these pre-treatment strategies with refined microfluidic architectures, DEP technology offers high specificity and label-free isolation, providing broad prospects for clinical biomarker detection (Wang et al., 2024a).

### 2.9 Summary of exosome extraction methods

**FIGURE 3:**
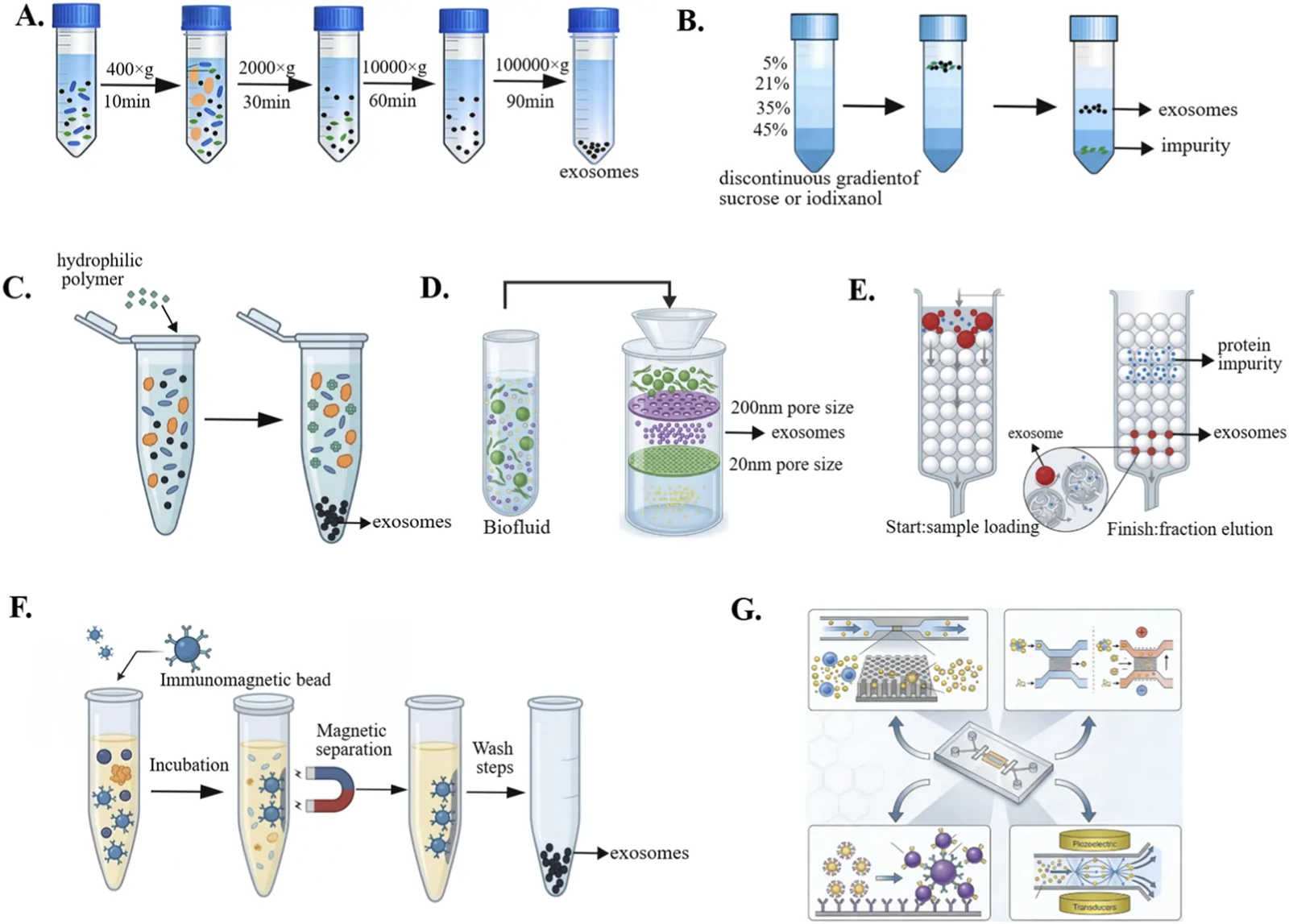
Schematic represenrtation of common exosomal separation techniques. **(A)** Differential centrifugation, **(B)** Density gradient centriugation, **(C)** Polymer precipitation, **(D)** Ultrafiltration, **(E)** Size exclusion chromatography, **(F)** Immunomagnetic separation, and **(G)** Microfluidics.

Table 1 summarizes eight common EVs isolation methods. Each of these methods has its own advantages and limitations and usually the appropriate method should be selected based on the downstream application and the type of sample, or a combination of multiple techniques. Schematic diagrams illustrating the principles of these exosome isolation methods are presented in Figure 3. Differential centrifugation serves as a foundational technique to obtain total EVs and is suitable for large-volume samples, such as cell supernatants and serum. However, it is time-consuming (typically 4–6 h) and yields EV preparations with low purity. To achieve high-purity EV preparations, it is recommended to use density gradient centrifugation or SEC. Density gradient centrifugation delivers the highest purity and is suitable for downstream applications with demanding exceptional purity (e.g., proteomic analysis). Notably, it is operationally complex, costly, and associated with low yield. SEC operates gently, preserves EV integrity and biological activity without reagent residue, and is suitable for serum, plasma and other biofluid samples, as well as functional assays that require maintaining complete biological activity. Polymer precipitation is easiest to implement and yields the highest recovery, but suffers from relatively low purity. It is suitable for rapid preliminary enrichment or functional assays with low purity requirements. For the isolation of specific EV subsets, immunoaffinity capture is the preferred approach. This method exhibits high specificity, enables the isolation of EV subsets from specific cellular origins, offers high purity, and is suitable for capturing EVs expressing specific markers (e.g., CD63, TSG101) for biomarker discovery. Nonetheless, it is technically complex and yields low recovery.

**TABLE 1:** Exosome isolation methods.

Exosome isolation methods	TIME (min)	Advantages	Disadvantages	Throughput	Refs
Differential centrifugation	240–360	Large sample volume No reagents required	Expensive instrument Repetitive steps damage	Medium	Welsh et al. (2024), Skotland et al. (2017), Lane et al. (2015), LAI et al. (2022), Livshits et al. (2015)
Density gradient centrifugation	600	Good purity	Time-consuming Complicated operation	Low	Vidal et al. (1997), Martins et al. (2023), Wu et al. (2025)
Polymer precipitation	30–120	Simple operation Time saving	Low purity Polymer residue	High	Yamada et al. (2012), Alvarez et al. (2012), Rekker et al. (2014), Zhu et al. (2020)
Ultrafiltration	130	Simple operation Wide range of sample volume	Low purity Reduced yield by flterclogging	High	Cheruvanky et al. (2007), Visan et al. (2022)
Immunomagnetic separation	240	High selectivity High purity for proteomicanalyses	Low sample volume Expensive Extra elution step	Low	Tauro et al. (2013), Da Fonseca Alves et al. (2024), Shao et al. (2020), Liu et al. (2024), Tauro et al. (2006)
Size exclusion chromatography	15	Good purity Preserves vesicle integrity	Protein contamination Complicated operation Dilution is required for viscous samples	Medium	Visan et al. (2022), Bing et al. (2014), Nordin et al. (2015), KIM et al. (2025)
Microfluidics	30–120	Low sample volume Good purity	Expensive Sample may clog the channel	Low	Fong et al. (2014), Feng et al. (2007), Liu et al. (2017), Kang et al. (2008), Hassanzadeh-BArforoushi et al. (2025), Verma et al. (2025a), Zhao et al. (2025)
Dielectrophoresis	30	Label-free and contactless Time saving	Low purity Low resolution	High	Luna et al. (2023), Hassanpour et al. (2021), Wang et al. (2024a)

### 2.10 Combinatorial strategies and recommendations

Recognizing that no single isolation method can simultaneously achieve maximum yield and absolute purity, combinatorial approaches are increasingly recommended by the Minimal Information for Studies of Extracellular Vesicles (MISEV) 2023 guidelines. To provide actionable guidance, we propose the following optimal combinations based on distinct downstream application scenarios (Welsh et al., 2024). For fundamental mechanistic and multi-omics studies (high purity priority): the combination of differential centrifugation followed by SEC (or density gradient) is highly recommended. Differential centrifugation concentrates the large-volume culture media, while SEC effectively eliminates co-isolated soluble proteins and lipoproteins, yielding highly purified EVs suitable for mass spectrometry and reliable *in vivo* functional assays (Chen et al., 2021). For biomarker discovery and clinical diagnostics (throughput and specificity priority): When working with limited clinical samples (e.g., patient plasma), microfluidics combined with immunoaffinity capture represents the optimal choice. As previously discussed, this approach allows for rapid, standardized isolation of specific EV subpopulations (e.g., TDEs) from minimal volumes, seamlessly integrating with downstream point-of-care analytical platforms (Abreu et al., 2022). For therapeutic applications and clinical translation (scalability priority): The transition from bench to bedside necessitates large-scale production. Here, TFF combined with SEC is the gold standard. TFF rapidly processes liters of conditioned media with high yield, while a subsequent SEC polishing step ensures the removal of process-related impurities, fulfilling the stringent safety and purity requirements for therapeutic EV preparations (Visan et al., 2022). The integration of Microfluidics and DEP for EV separation and detection offers advantages of facile control, high specificity, and high sensitivity (Wang et al., 2024b).

## 3 Exosome components

### 3.1 Protein

Nearly 10,000 different proteins exist in exosomes of different origins, and owing to their high expression levels, many have been widely used as biomarkers. Exosomes from different sources have similar biosynthetic pathways, so most proteins contained in exosomes are related to the MVB formation process. Almost all exosomes contain some membrane proteins, such as CD9, CD63, CD81, which serve as markers to distinguish exosomes from other vesicles (Vlassov et al., 2012). Other membrane proteins are cell-specific and unique to exosomes secreted by particular cell types. These membrane proteins are mainly used to isolate exosomes derived from specific cells. Examples include A33 (derived from colonic epithelial cells), lectin (derived from immature dendritic cells), and CD1, CD86, MHC I, and MHC II from T cells (Poliakov et al., 2009). Exosomes also contain abundant inner membrane proteins. The most abundant proteins are associated with exosomal biological functions. Exosomes contain cytoplasmic proteins, for example, RAB proteins promote the fusion of MVBs with cell membranes and the release of exosomes; TSG101, CHMP4, syntenin and the accessory protein Alix participate in the formation of ESCRT and promote inward budding of endosome membranes. There are also heat shock protein families (HSP60, HSP70, HSPA5, CCT2 and HSP90), of which HSP70 and HSP90 promote the binding of antigen peptides to MHC I, and MHC II (Cho et al., 2005). They also harbor signal transduction proteins such as G protein and protein kinases (Mathivanan et al., 2012), as well as signal transduction factors (melanoma differentiation-related factors, ARF1, CDC42), metabolic enzymes (GAPDH, enolase 1, aldolase 1, PKM2, PGK1), and ribosomal proteins (RPS3). Exosomes contain abundant proteolytic enzymes, suggesting that exosomes can promote cell metastasis. Exosomes also contain adhesion factors (MFGE8, integrin), cytoplasmic tubulin, cytoskeleton proteins including actin, myosin, tubulin, actin-binding protein, and ubiquitin. Exosomes secreted by tumor cells can carry specific proteins, which are helpful for the diagnosis and detection of tumors. Studies (Ataysafinurwilkey et al., 2018) have shown that exosomes secreted by intestinal tumors are rich in diagnostic markers such as KIT, CD34, ANO1, PROM1, PRCC, and ENG.

To date, the majority of studies have centered on profiling exosomal protein cargos in various biological fluids and tumor models, yielding notable advances in identifying candidate diagnostic biomarkers for malignant diseases. Nevertheless, key knowledge gaps persist in the field: first, the functional interplay among distinct exosomal protein subsets (e.g., ESCRT machinery, HSPs, and metabolic enzymes) remains poorly characterized, with little mechanistic evidence connecting protein composition to exosomal biological activity; second, the selective sorting processes governing tumor-specific protein enrichment in exosomes are not yet fully understood, limiting the rational design of targeted exosome isolation approaches (Welsh et al., 2024).

### 3.2 Nucleotide

The abundance of nucleotides in exosomes is a feature that distinguishes them from other biological vesicles. The nucleotides in exosomes mainly consist of RNAs, including mRNA, miRNA, lncRNA, piRNAs, ribosomal RNA, snRNA, tRNA mitochondrial RNA. When the nucleotides in exosomes are transferred to target cells, they can be translated into functional proteins that affect the synthesis and expression of proteins in recipient cells. Exosomal miRNAs function as central regulators of vital biological phenomena, such as inflammatory signaling, fibrogenesis, and wound healing. Given their regulatory prominence, they are increasingly recognized as viable therapeutic focal points. The deployment of synthetic or modified EVs integrated with specific miRNA mimics or inhibitors allows for the targeted manipulation of these pathways, thereby fostering tissue regeneration and impeding the evolution of malignancy or chronic conditions (Verma et al., 2025b). Exosomal miRNAs, such as let-7, miR-1, miR-15, miR-16, miR-151, and miR-375, play important roles in angiogenesis, exocytosis, and tumor development (Taylor et al., 2011). Exosomes containing miRNA-205 derived from human bone marrow mesenchymal stem cells can prevent the progression of prostate cancer (Jiang et al., 2019). Exosomal nucleic acids can be stably present in blood, urine and other body fluids due to the protection of the exosomal membrane. The parental cell-specific components in exosomes can be used to trace the presence of their cells of origin. Therefore, exosomal miRNAs can be used as biomarker to aid clinical diagnosis of diseases. For example, exosomal miRNAs (such as let-7a, miR-1229, miR-1246, miR-150, miR-21, miR-223 and miR-23a) can be used as biomarkers for the diagnosis of colorectal cancer (Ogata-kaw et al., 2014). LncRNAs in exosomes also play an important role in the occurrence and progression of cancer. Exosome-transmitted lncRNA H19 Inhibits the growth of pituitary adenoma.

Exosomes also contain several types of DNA, including single-stranded DNA (ssDNA), double-stranded DNA (dsDNA) (Kahlert et al., 2014), mitochondrial DNA (mtDNA) (Sansone et al., 2017), and genomic DNA (Kalluri and Lebleu, 2020). Exosomal DNA can be transferred to recipient cells, where it plays a transient role. Studies have shown that treatment with epidermal growth factor receptor (EGFR) (Montermini et al., 2015) or topoisomerase inhibitors (Kitai et al., 2017) increases DNA packaging in exosomes, but the exact mechanism controlling DNA packaging in exosomes remains unclear.

### 3.3 Lipids

Compared with protein and nucleotide studies, studies on exosome lipids are relatively rare. The lipids of exosomes include sphingolipids, cholesterol, diacylglycerols, synglucoside GM3, saturated fatty acids, phosphoacylserine and ceramide. Compared with the cell membrane of the parental cell, the contents of phosphocholine and dionyl glycerin on the surface of the exosome membrane were decreased (Laulagnie et al., 2004). However, the surface of exosomes is rich in lysobisphosphatidic acid (LBPA), which facilitates the internalization by recipient cells and plays an important role in the biological functions of exosomes (Fitzner et al., 2011). Lipids in exosomes can participate in the biogenesis of exosomes and regulate homeostasis in recipient cells (Minciacchi et al., 2015). Lipids rich in MVBs, such as LBPA, lead to the formation of ILVs, and the interaction between LBPA and Alix promotes the budding of MVBs membranes (Bissig et al., 2013). Acidic and neutral sphingolipases can generate sphingolipid ceramides that drive the biogenesis of ILVs within MVBs (Verderio et al., 2018). Flaherty et al. reported (Flaherty et al., 2019) that adipocyte-derived exosomes can be internalized by macrophages in adipose tissue to regulate the differentiation and function of these tissue macrophages. Phospholipase D and phosphatidic acid play important roles in the biogenesis and cargo loading of EVs (Luis and Pascale, 2018). The lipids carried by exosomes can also be used as an important bioactive molecules involved in diverse biological processes, including immune surveillance, tumor microenvironment (TME) remodeling and inflammation regulation (Record et al., 2018). For example, tumor-derived exosomes (TDEs)rich in PGE_2_ can remodel the TME into a tumor-permissive microenvironment.

Taken together, the above components of exosomes are summarized in the following figure (Figure 2).

## 4 Biogenesis and secretion of exosomes

### 4.1 Biogenesis of exosomes

The biogenesis of exosomes is a sophisticated, multi-step process tightly regulated by various molecular mechanisms and signaling pathways, which mainly consists of three core sequential steps: the formation of ILVs, the transport of MVBs, and the fusion of MVBs with the plasma membrane. Initially, the plasma membrane undergoes invagination triggered by endocytic processes (including clathrin-dependent endocytosis, caveolin-mediated endocytosis, and clathrin/caveolin-independent endocytosis), leading to the formation of small membrane-enclosed vesicles that subsequently mature into early endosomes (EE). These EEs are then integrated into the endosomal recycling system, where they undergo extensive cargo sorting—selectively encapsulating intracellular components such as proteins (e.g., tetraspanins, heat shock proteins), lipids, nucleic acids (mRNA, miRNA, circRNA), and metabolites (van Niel et al., 2018). During the maturation process of EE into late endosomes (LE), the endosomal membrane undergoes profound structural changes, characterized by inward budding and invagination, which further drives the formation of ILVs. Notably, these ILVs, which are destined to become exosomes upon secretion, encapsulate the sorted cargoes in a highly selective manner, and the endosome filled with numerous ILVs is morphologically defined as an MVB.

**FIGURE 4:**
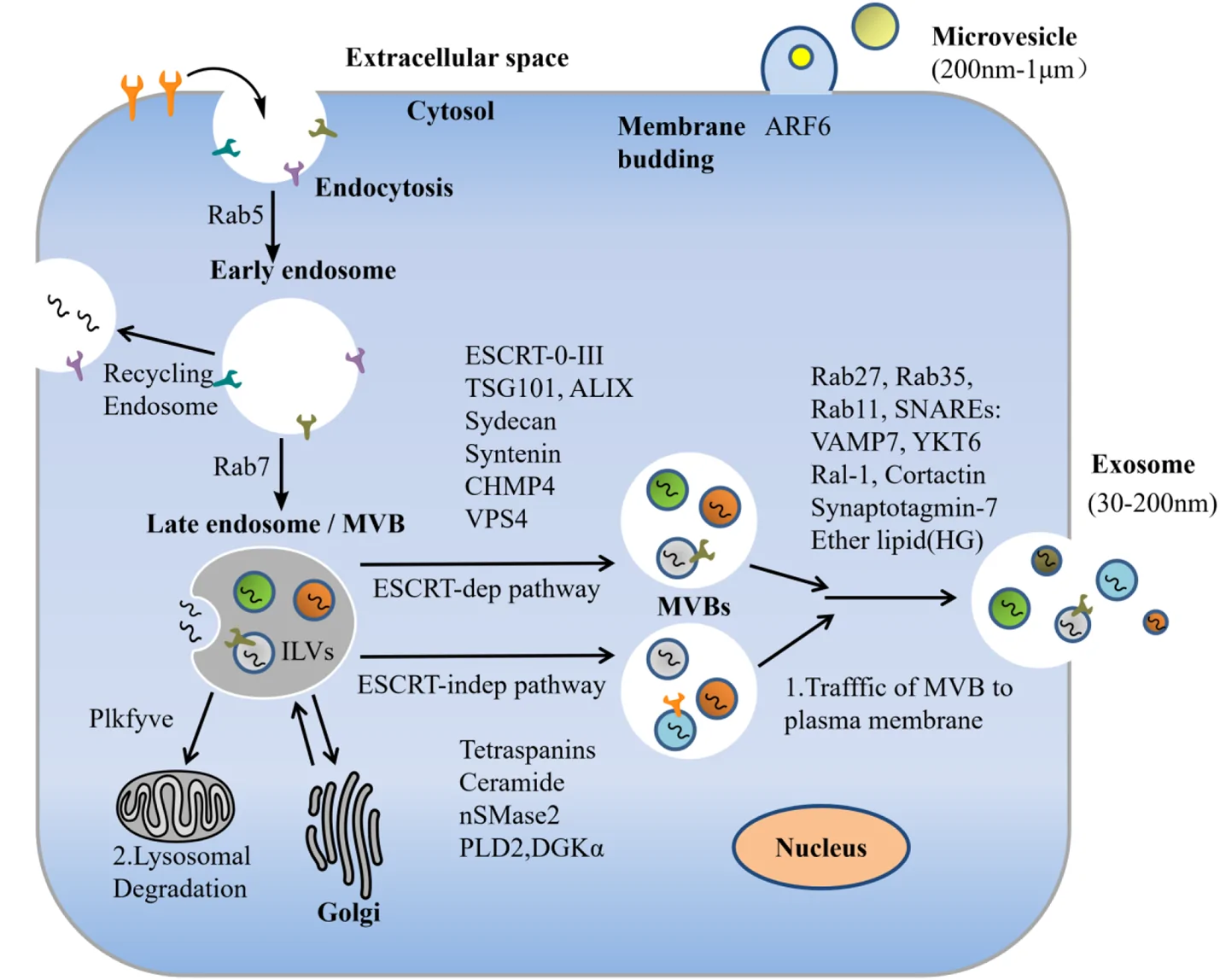
Schematic overview of the molecular mechanisms governing exosome biogenesis and secretion. Exosomes are formed as intraluminal vesicles (ILVs) within multivesicular bodies (MVBs) via parallel endosomal pathways. Cargo sorting into ILVs is orchestrated by an ESCRT-dependent mechanism (involving ESCRT-0-III, ALIX, TSG101, and VPS4) and a distinct ESCRT-independent mechanism (driven by tetraspanins and lipid enzymes like nSMase2). Post-maturation, MVBs face a fate decision between lysosomal degradation or trafficking to the cell membrane. Traffic and subsequent docking/fusion of secretion-fated MVBs are controlled by a coordinated machinery of Rab GTPases (e.g., Rab27a/b, Rab11, Rab35), Ral-1, and specific SNARE complexes (e.g., VAMP7, YKT6), culminating in exosome release. Conversely, microvesicles form through direct outward budding of the plasma membrane, regulated by ARF6. The step of exosome biogenesis and secretion affected, or likely to be affected, by each molecule is displayed on the figure.

There are two main fates of MVBs formed, one is the fusion of MVBs with autophagosome or lysosome, and the final degradation of its contents. The degradation products can be recycled by cells. The other is the fusion of MVBs with cytoplasmic membrane, which releases the contained ILV as exosomes into the extracellular environment (Kahlert and Kalluri, 2013). The levels of cholesterol in the MVBs seems to determine their fate, cholesterol-rich MVBs fuse with the plasma membrane to release exosomes, whereas another less cholesterol-rich MVBs fuse with lysosomes to degradate (Mobius et al., 2002). The molecular mechanisms underlying exosome biogenesis are primarily classified into two distinct pathways: endosomal sorting complex required for transport (ESCRT)-dependent and ESCRT-independent pathways, both of which cooperate to regulate ILV formation and MVB fate, albeit through different molecular machineries (Figure 4). Detailed elaboration of these two pathways is provided as follows.

### 4.2 The mechanism of ESCRT dependence

The function of ESCRT during exosome biogenesis was originally proposed from proteomic studies (Thery et al., 2001). ESCRT contains four complexes and associated auxiliary proteins, ESCRT-0, ESCRT-I, ESCRT-II, ESCRT-III, and associated AAA ATPase VPS4, each orchestrating different steps in cargo sorting and exosome biogenesis. Subunits of ESCRT-0 include HRS, STAM1, and STAM2. HRS recognizes ubiquitin-labeled proteins and interacts with STAM (Ren and Hurley, 2010). HRS and STAM collectively contain up to 10 ubiquitin-binding sites in total, so ESCRT-0 is ideal for capturing polyubiquitin cargos. HRS is a cytoplasmic protein that targets early endosomes by binding its FYVE domain to phosphatidylinositol 3-phosphate (PtdIns3P). Subsequently, HRS binds to clathrin and the ESCRT-0 complex is recruited to cluster and capture ubiquitinated cargo (Vietri et al., 2019). ESCRT-0 hands over the ubiquitinated cargos to ESCRT-I. Then, ESCRT-I recruits ESCRT-II, which contains a ubiquitin-binding and PtdIns3P-binding GLUE domain. Finally, ESCRT-III is recruited by ESCRT-II to nucleate CHMP2-4 polymers into membrane-associated filaments, which cooperate with the AAA ATPase Vps4 to mediate ILV sculpting and scission. The ESCRT machinery is recruited by the proteoglycan syndecan via the small adaptor protein syntenin-1 and the ESCRT-binding protein ALIX, syntenin-1 interacts with a LYPX(n)L motif in ALIX, which in turn binds to the cytosolic tail of syndecan. Previous literature has been reported that ESCRT-II and the ESCRT-III subunit CHMP6 cooperate with ESCRT-I to recruit CHMP4B (Christ et al., 2016). However, the recruitment of CHMP4C relies predominantly on ALIX.

The proteins involved in exosome biogenesis play an important role in the synthesis and secretion of exosomes. The amount of these proteins can directly or indirectly affect the amount of exosomes. Colombo M et al. showed that inhibition of the ESCRT-0 proteins Hrs, or depletion of TSG101 and the ESCRT-I protein STAM1 reduced the secretion of exosomes. In contrast, VPS4B knockdown increased exosome secretion, the roles of these four proteins were analyzed by Western blot (WB) and verified in exosomes isolated via ultracentrifugation (Colombo et al., 2013). In addition, the depletion of TSG101, an ESCRT-I member, can also reduce the secretion of exosomes (Abrami et al., 2013). It has been found in breast cancer cells that the absence of CHMP4 proteins (ESCRT-III members) reduces exosome secretion (Baietti et al., 2012).

Exosome biogenesis-associated proteins can exert pleiotropic effects on exosome production in different cellular environments. The auxiliary protein ALIX promotes endosome sprouting to form intraluminal vesicles, thereby increasing exosomal secretion (Baietti et al., 2012). However, in HeLa-CIITA cells, knockout of ALIX can increase the accumulation of MHC class domains in the cytoplasm and has no effect on exosomal biogenesis, (Colombo et al., 2013). Studies have reported that VPS4 is involved in the final step of intraluminal vesicle formation, facilitating the separation of membranes from ESCRT-III complexes (Hanson and Cashikar, 2012). In HeLa-CIITA cells, Inhibiting the function of VPS4B can increase exosomes secretion (Colombo et al., 2013). However, in breast cancer cells, simultaneous silencing of VPS4A and VPS4B increases exosome biogenesis, whereas silencing of either VPS4A or VPS4B has no significant effect on exosome biogenesis (Baietti et al., 2012). In RPE1 cells, simultaneous inhibition of VPS4A and VPS4B can reduce exosome secretion (Abrami et al., 2013). In summary, each of these proteins plays an important role in exosomes biogenesis. These studies fully demonstrate that although the ESCRT pathway is crucial for exosome biogenesis and secretion, the overall process is highly complex.

### 4.3 The mechanism of ESCRT independence

Some studies have shown that in addition to the classic ESCRT-dependent mechanism, cells can also generate ILVs and MVBs through ESCRT-independent mechanism, mainly including lipids, tetraspanins transmembrane proteins, and heat-shock proteins (HSP). Trajko et al. (2008) confirmed the ESCRT-independent mechanism of exosomal biogenesis for the first time. They silenced the four complexes of ESCRT simultaneously in an experimental model and found that exosomes containing proteolipid protein (PLP) can still form normally. Ceramides produce exosomes in an ESCRT-independent mechanism. Neutral sphingomyelinase (nSMase) is an enzyme that produces ceramide by hydrolyzing sphingomyelin (Figure 4). Further research found that in these several cell lines (Kosaka et al., 2010; Chairoungdua et al., 2010), inhibition of the enzyme can reduce the production of ceramide, which in turn reduces the inward budding of the MVB membrane and reduces the formation of exosomes. However, in PC-3 cells, nSMase2 Neither inhibition of ceramide nor *de novo* synthesis of ceramide affects exosomal release (Phuyal et al., 2014). Besides, phospholipase D2 (PLD2) can hydrolyze phosphatidylcholine to phosphatidic acid. Studies have reported that the formation of exosomes requires the participation of PLD2 (Ghossoub et al., 2014).

Tetraspanins protein are enriched transmembrane proteins in exosomes and are also involved in the release of escrt-independent exosomes. In human melanoma cells, CD63 can sort melanin proteins into ILV. This process does not rely on the ceramide pathway or the ESCRT pathway. Knockout CD63 with CRISPR/Cas9 can reduce secretion of EVs (Hurwitz et al., 2016), and CD63 is required for LMP1-induced particle secretion and packaging into exosomes. TSPAN8 (Nazarenko et al., 2010) and CD81 (Perez-Hernandez et al., 2013)can also sort a series of ligands into exosomes. The protein of tspan8 does not affect the total secretion of exosomes, but changes the mRNA and protein composition of exosomes, in rat adenocarcinoma cells. In HEK293 cells, overexpression of the tetraspanins protein CD9 or CD82 can promote exosome biogenesis (Chairoungdua et al., 2010).

In addition, the chaperone hsp70 can recruit the transferrin receptor (TFR) to exosomes and bind the cytoplasmic protein KFERQ to transport the cytoplasmic protein to ILVs (Sahu et al., 2011). Both mechanisms of exosome biogenesis, as described above, are important, but the pathways may not be completely isolated. These pathways may work together, and different exosome subsets may depend on different mechanisms. With the development of research, more experiments are needed to fully confirm the functional effect of ESCRT independent mechanism to assist exosome generation.

### 4.4 Secretion of exosomes

Besides the regulation mechanism of exosome biogenesis, the regulation of exosome secretion is also an important scientific problem. After the formation of MVBs in cells, different subgroups of MVBs have different degradation pathways. The secreted MVBs fuse with the plasma membrane to release exosomes, while the degraded MVBs fuses with lysosomes, resulting in the degradation of MVBs and their concents. Rab protein is the largest subfamily of Ras superfamily of GTPase binding proteins, which can regulate different stages of vesicle transport, such as vesicle budding, vesicle movement and vesicle connection, thus promoting membrane fusion. As an intracellular transport protein, Rab protein plays an important role in the regulation of endocytosis and exocytosis (Stenmark, 2009). The first Rab protein reported to be related to exosome secretion was Rab11. In human leukemia K562 cells, Rab11 can regulate exosome secretion. Overexpression of the Rab11 mutant plasmid reduces exosome secretion (Savina et al., 2002). Still another Rab, Rab35 regulates exosome release by controlling the docking/tethering of endocytic vesicles with the plasma membrane, and inhibiting the expression of Rab35 can reduce exosomal secretion in neutrophils and primary oligodendrocytes. Subsequently, it was also confirmed that exosome secretion required the involvement of Rab11 and Rab35 in human RPE1 cells (Abrami et al., 2013). The role of Rab27 in exosome release has been reported in tumor cells. Studies have shown that exosome secretion is reduced in melanoma and breast cancer cells when Rab27A silenced. In human breast cancer cells, the deficiency of Rab27A did not reduce exosome secretion, but the simultaneous deletion of Rab27A and Rab27B reduced exosome secretion, in human breast cancer cells (Zheng et al., 2013). In HeLa cells, silencing Rab2B, Rab5A, Rab9A, Rab27A, and Rab27B can all reduce exosome secretion. The deletion effect of Rab27A and Rab27B is more obvious, while the absence of Rab11A or Rab7 has no the secretion of exosomes (Ostrowski et al., 2010). However, in McF-7 cells, Rab7 is required in secretion of exosomes containing syntenin and ALIX (Baietti et al., 2012). In addition, Small GTPases of other families such as the Rho/Rac/cdc42 family may also play an important role in exosome release. In particular, the RhoA effector citron kinase was shown to increase exosomal release, in HIV-1 virion (Loomis et al., 2006). These different findings may be due to the heterogeneity of the cells, or due to different methods of isolating vesicles and quantifying the release of exosomes.

In addition to Rab proteins, the soluble NSF-attachment protein receptor (SNARE) family is another important regulatory molecule in exosome secretion. It is a complex composed of multiple proteins that facilitates the fusion of lipid bilayers after precise docking of 2 cells carrying different components, which involves one R-SNARE (usually v-SNARE) and three Q-SNAREs (usually t-SNAREs). In different types of cells, the SNARE proteins SNAP-23, VAMP-7 and VAMP-8 are involved in the fusion of lysosomal secretions regulated by calcium ions and cell membranes. Study have shown that overexpression of the N-terminal domain of vesicle-associated membrane protein 7 (VAMP7), a R-SNARE protein, which inhibits SNARE complex formation and the fusion of MVBs with the plasma membrane, reduced exosome release (Fader et al., 2009). Another R-SNARE protein, Ykt6 promotes secretion of exosomes loaded with morphogen WNT3A, TSG101 and VPS26/35 in human embryonic kidney HEK293 cells (Gross et al., 2012), however, there is no evidence that it affects MVE fusion and exosomal release directly. Recently, pyruvate kinase type M2 (PKM2) was shown to phosphorylate SNAP-23, which can promote tumour cell exosome release (Wei Y. et al., 2017). Syntaxin 1A (STX1A) has also been reported to act on exosomes secretory mechanism, inhibition of the Q-SNARE syntaxin 1A (Syx1A) reduced release of Evi-bearing exosomes, in *drosophila* S_2_ cells (Koles et al., 2012). Ral-1 (Ras-related GTPase) exists on the surface of MVBs membrane and is related to the formation of MVBs and the fusion of MVBs and cytoplasmic membrane. Synaptic fusion protein 5 (STX5), a downstream protein of Ral-1, also plays an important role in the fusion of MVBs membrane and cytoplasmic membrane. Knocking out STX5, the MVBs membrane no longer fuses with the cytoplasmic membrane, allowing MVBs to accumulate in the cell (Hyenne et al., 2015). At the same time, the process requires the participation of RalA and RalB. Overexpression of diacylglycerol kinase (DGKa) in T cells can inhibit exosome secretion. In MCF-7 cells, Rab3D was found to promote exosome secretion (Yang et al., 2015). In short, there may be many effector molecules in cells that are related to exosome secretion regulation. Transmembrane secretion, extracellular secretion and other pathway mechanisms need to be further explored, which will help comprehensive understanding of exosomes.

## 5 The role of exosomes in tumor progression

As a natural intercellular information carrier, exosomes have great application potential in the field of drug carriers due to their relatively small molecular structure, natural molecular transport properties, and good biocompatibility. Using exosomes as a drug carrier can not only enhance the antitumor effect of the drug, but also reduce the adverse reactions of the drug to the main system or organ. Tumor cells secrete more exosomes than normal cells. Once exosomes fuse with the target cell’s plasma membrane, they release the contents, which mediates the transmission of signals and substances between the tumor and various cells, and realize the communication between cells in the TME. As a carrier of intercellular communication between cells, exosomes can transfer the bioactive substances such as protein nucleic acid, which can not only regulate the proliferation and apoptosis of tumor cells, but also regulate the microenvironment of tumor cell growth. Therefore, exosomes are extensively involved in tumor occurrence, development, metastasis, immune avoidance and drug resistance of chemotherapy drugs (Kalluri and Lebleu, 2020).

### 5.1 Exosomes in cancer cell migration and invasion

**FIGURE 5:**
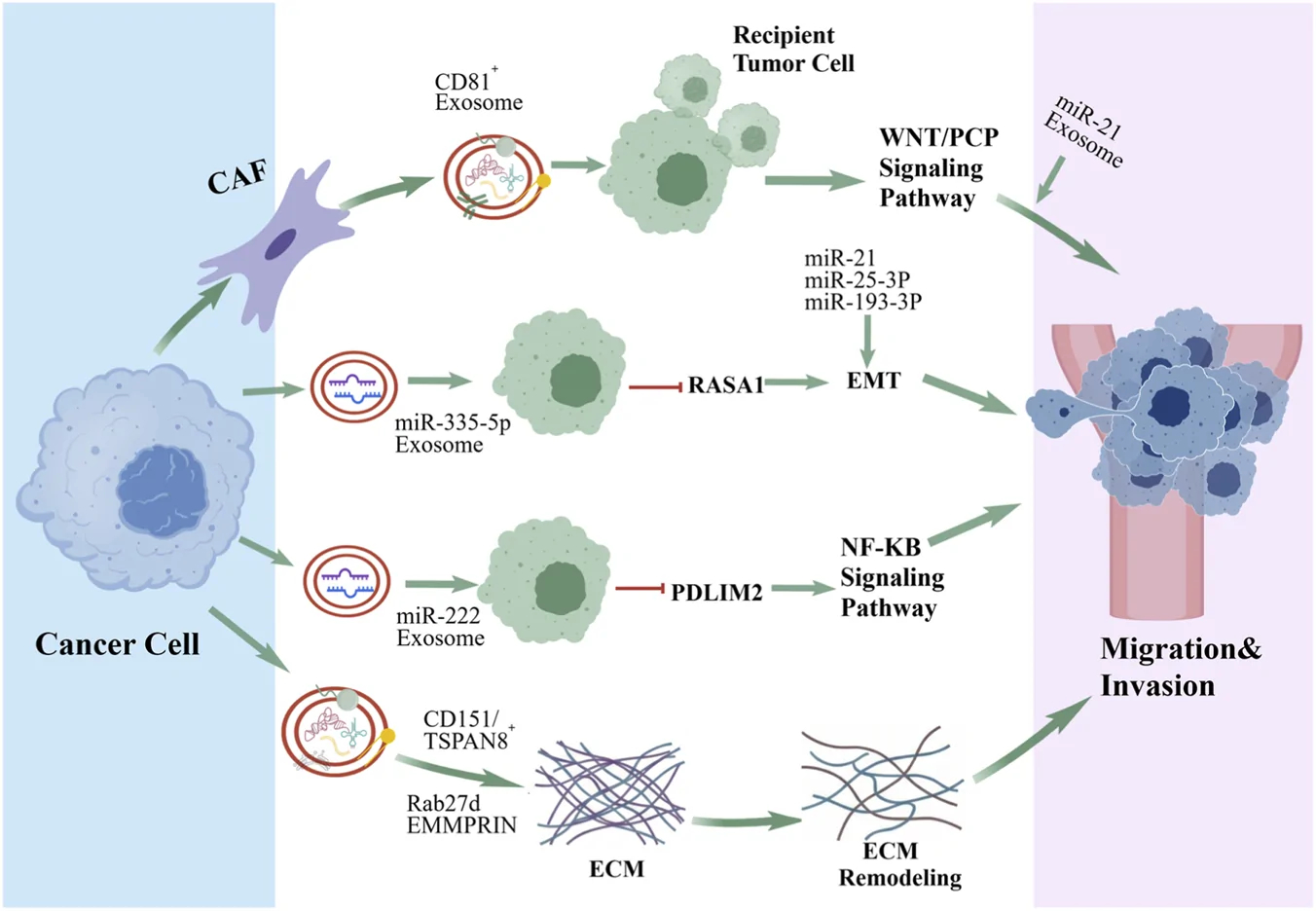
Exosomal regulation of signaling pathways and microenvironmental remodeling in cancer metastasis. The diagram depicts the multifaceted role of exosomes in the primary tumor microenvironment and metastatic progression. (Top) Cancer-associated fibroblasts (CAFs) secrete CD81^+^ exosomes to activate the WNT/PCP signaling pathway in recipient tumor cells. Additionally, miR-21 exosomes act directly to facilitate distant metastasis. (Middle) Tumor-derived exosomes (TDEs) transfer specific miRNAs to regulate intracellular signaling. Exosomal miR-335-5p inhibits RASA1, while a cluster of miRNAs (miR-21, miR-25-3p, miR-193-3p) collectively promotes Epithelial-Mesenchymal Transition (EMT). Simultaneously, miR-222 exosomes downregulate the tumor suppressor PDLIM2, activating the NF-κB signaling pathway. (Bottom) Exosomes enriched with CD151, TSPAN8^+^, Rab27d, and EMMPRIN interact with the extracellular matrix (ECM), driving ECM remodeling. These synergistic pathways enhance tumor cell migration, invasion, and subsequent metastatic colonization.

During the process of cancer development, normal cells gradually evolve into malignant lesions, which eventually lead to the formation of local malignant tumors, followed by distant metastases. Tumors occur when cells acquire transformation mutations of cancer driver genes through genetic or sporadic events (Levine et al., 2020). Tumor metastasis refers to the process in which malignant tumor cells spread from the primary site through lymphatic channels, blood vessels or body cavities to other sites and continue to grow. It is the main cause of cancer treatment failure. In 2012, It has been reported that cancer-associated fibroblasts (CAFs) secreted CD81-positive exosomes, promoted the invasion, motility and metastasis of breast cancer cells by activating the autocrine WNT/PCP signaling pathway (Luga et al., 2012). TDEs can promote cell invasion and metastasis by regulating the extracellular matrix. For example, CD151/TSPAN8-positive exosomes derived from the rat pancreatic adenocarcinoma cell line (ASML) influenced the activation of hematopoietic cells and stromal cells by regulating the remodeling of the host cell matrix (Yue et al., 2015). TEMs can promote the epithelial-mesenchymal transition (EMT) of receptor cells, and facilitate the invasion and metastasis of tumors. The latest research indicates that exosomal miR-335-5p, derived from the metastatic colorectal cancer cell line SW620, promotes EMT by targeting RAS p21 protein activator 1 (RASA1) to promote invasion and metastasis of colorectal cancer cells (Sun et al., 2021). It is reported that exosomes derived from A549 cells and H460 cells significantly enhanced the EMT of lung tumor cells and promoted the growth of subcutaneous xenograft tumors (Zhiyuan Zhoua et al., 2022). TDEs containing miR-222 promoted the migration and invasion of breast cancer cells through downregulation of PDLIM2- (a tumor suppressor) which lead to activation of NF-κB signaling pathway (Wei F. et al., 2017) (Figure 5).

### 5.2 Exosomes in tumor angiogenesis

Tumor angiogenesis is of vital importance for the growth and metastasis of tumors, as it can provide sufficient oxygen and nutrients for tumor development. TDEs regulate the tumor angiogenesis process through multiple pathways and targets in the TME by transporting and delivering a variety of tumor angiogenesis factors. TDEs promote angiogenesis by delivering miR-130a or miR-155 from gastric cancer cells to human endothelial cells (Deng et al., 2020), TDEs can regulate endothelial cells to stimulate tumor angiogenesis. He et al. (2019) discovered that exosomal miR-205 secreted by ovarian cancer induces tumor angiogenesis by regulating the PTEN-AKT signaling pathway. Exosomes from colorectal cancer carry miR-25-3p, which are transferred from cancer cells to endothelial cells and target the transcription factors KLF2 and KLF4, regulating the expression of VEGFR2, ZO-1, Oc-cludin and Claudin5 proteins in endothelial cells, thereby enhancing vascular permeability and promoting tumor angiogenesis (Ma et al., 2021; Wang et al., 2020; Wu et al., 2019). TDEs enriched with TSPAN8 and integrin subunit α4 can upregulate angiogenesis-related genes to enhanced endothelial cell proliferation and angiogenesis (Research N. J. C., 2010). TDEs of circRNAs can accelerate the metastasis process of tumors by promoting tumor angiogenesis. Huang et al. (2020) discoved that exosomal circRNA-100338 promoted the growth, migration and angiogenesis of endothelial cells, and enhanced vascular permeability and the migration of liver cancer cells by reducing the expression levels of vascular endothelial cadherin and ZO-1 in HUEVC cells.

### 5.3 Exosomes and the TME

TME is a complex and dynamic network composed of malignant tumor cells, stromal cells, various immune and inflammatory cells, fibroblasts, and angiogenic factors. It is the intrinsic environment for tumor growth, proliferation, invasion, and metastasis (Barkley et al., 2022). Exosomes have been identified as an important medium for communication between tumor cells and the TME, and they serve as an important carrier of cell-to-cell communication. Studies have shown that the circPVT1 carried by exosomes derived from non-small-cell lung cancer (NSCLC) A549 cells mediates the M2 polarization of macrophages through the miR-124-3p/EZH2 axis, thereby inhibiting immune function and enhancing the proliferation, invasion and migration of tumor cells (Liu et al., 2022). Exosomes and TME are closely linked, as the signaling pathways affected by exosomes can influence TME. TDEs can regulate the TME and extracellular matrix (ECM) by stimulating extracellular receptor signal transduction and disrupting cell adhesion formation. Studies have shown that various integrins and integrin ligands can be carried by TDEs. Exosomal integrins are involved in initiating cancer cell colonization and formation of the pre-metastatic microenvironment (Paolillo and Schinelli, 2017). Additionally, exosomes play a crucial role in ECM remodeling and TME reprogramming, TDES induce differentiation of many types of TME cells to CAFs that are dominant cell population of the TME in most cancers (Zhang and Peng, 2018). Gemcitabine treatment of CAFs in pancreatic cancer induce the expression of Snail and miR-146a, prolong exosome secretion which stimulates epithelial cell proliferation (Richards et al., 2017). CAFs have been reported to metastasize exosomes into lung cancer cells, inhibit the killing of peripheral blood mononuclear cells (PBMCs) by upregulating PD-L1, and promoting the progression of lung cancer through the OIP5-AS1/miR-142-5p/PD-L1 axis (Jiang et al., 2021).

### 5.4 Exosomes in tumor immunity

**FIGURE 6:**
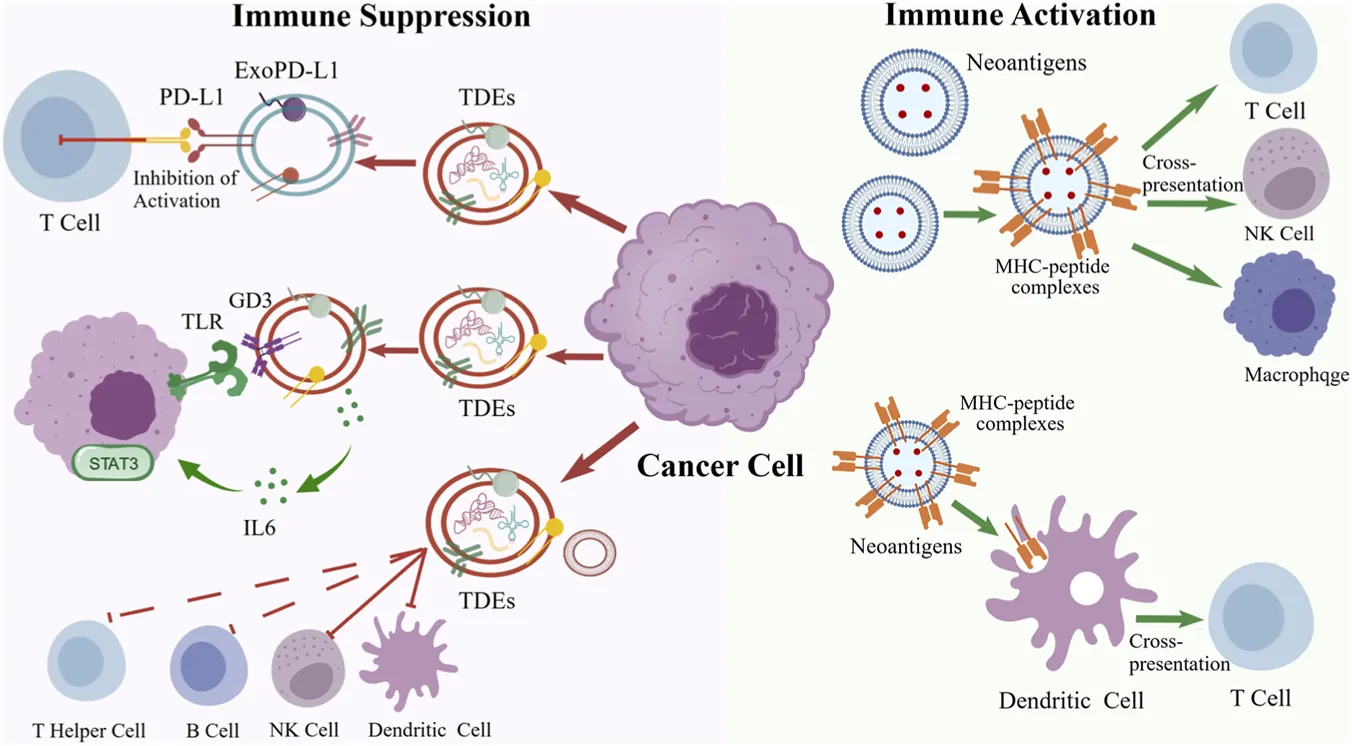
The dual immunomodulatory roles of tumor-derived exosomes (TDEs) in the tumor microenvironment (TME). Tumor cells secrete heterogeneous TDEs that mediate contrasting immune responses. Left panel (Immune Suppression): TDEs promote an immunosuppressive environment through multiple pathways. Exosomal PD-L1 (ExoPD-L1) binds to the PD-1 receptor on T cells, directly inhibiting T cell activation. Additionally, exosomal GD3 gangliosides interact with Toll-like receptors (TLR) on myeloid cells, triggering the release of IL-6 and subsequent activation of the STAT3 signaling pathway. TDEs also impair the functions of T helper cells, B cells, NK cells, and dendritic cells (DCs). Right panel (Immune Activation): Conversely, TDEs can elicit anti-tumor immunity. TDEs carrying neoantigens and MHC-peptide complexes can directly activate T cells, NK cells, and macrophages. Alternatively, TDEs are internalized by DCs, facilitating antigen cross-presentation to prime and activate T cells. Green arrows indicate activation or stimulation; red dashed lines indicate inhibition.

Exosomes can assist tumor cells to achieve immune escape and promote tumor invasion and metastasis. Studies have shown that prostate cance cells released PD-1 in exosomes that boosted the activity of myeloid-derived suppressor cells (MDSCs) by stimulating JAK/STAT3 signaling. The activated MDSCs in turn suppressed the infiltration of CD8^+^ T cells within the tumor, facilitating tumor immune evasion (Jie Zhang et al., 2025). Exosomes in ovarian cancer cells can bind to T cell receptors and induce T cell arrest, and exosomal GD3 binds to Toll receptors to activate IL-6 and act on the STAT3 pathway to promote immune escape of cancer cells (Wang et al., 2021). TDEs can carry PD-L1, namely, ExoPD-L1, in the TME, it can replace the cell surface PD-L1 and inhibit the activation of T cells, thereby creating suitable conditions for the growth of tumor cells and forming an immunosuppressive TME (Ye et al., 2021). ExoPD-L1 inhibits T-cell immune responses through multiple pathways, which can be summarized as directly or indirectly binding to PD-1 on the surface of T cells to inhibit T-cell activation, mediating the immune escape of tumor cells, and ultimately promoting tumor progression (Zhang and Zhang, 2020). Exosomes can also inhibit the immune function of other lymphocytes, such as T helper cells (Ye et al., 2016), B cells (Li et al., 2015), dendritic cells (DCs) (Tesone et al., 2013), and NK cells (Whiteside and Transactions, 2013). In addition, TDEs have been reported to activate immune responses. TDEs present neo-antigens and MHC-peptide complexes to launch and activate T cells by direct presentation and cross-presentation through DCs, or directly activate NK cells or macrophages (Filipazzi et al. 2012; Hao et al., 2022) (Figure 6).

EV-miRNAs act as pivotal regulators of tumor immunity by targeting core signaling pathways. For instance, EV-miRNAs can modulate the NF-κB and IRAK1/TAB2 axes to suppress anti-tumor inflammatory responses, or regulate the PI3K/Akt and TGF-β/SMAD pathways to alter immune cell polarization and tumor microenvironment remodeling. These mechanistic insights, validated in EV-mediated intercellular communication, strengthen the rationale for our focus on miRNA cargo in tumor immune evasion.

### 5.5 Exosomes in therapy resistance

**FIGURE 7:**
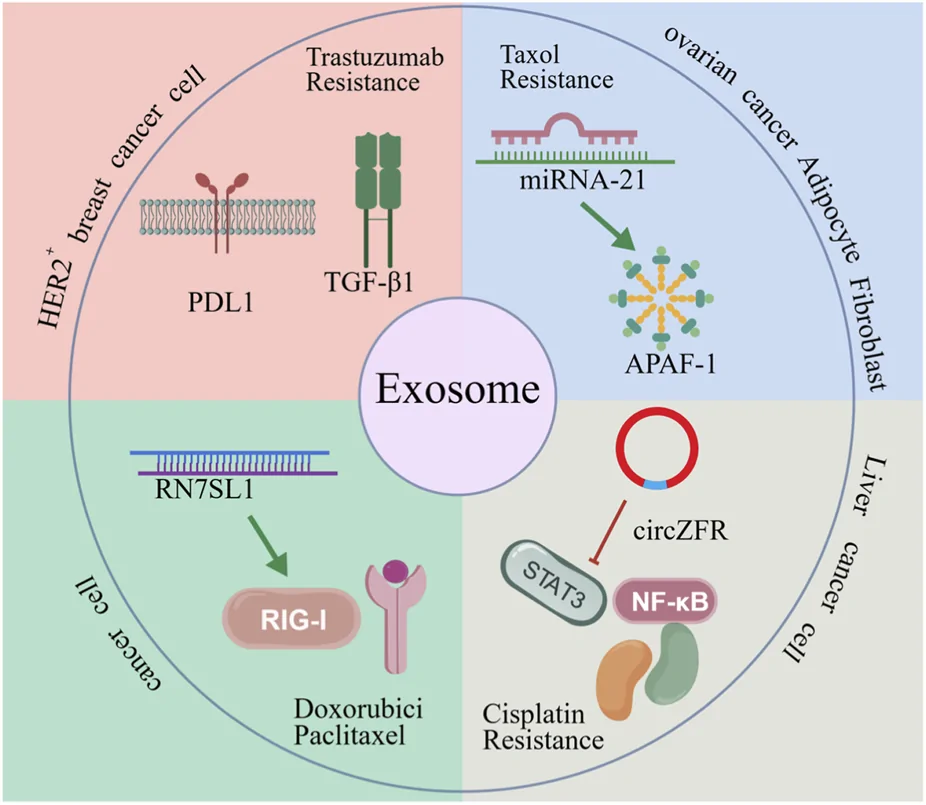
Schematic representation of exosome-mediated drug resistance mechanisms in the tumor microenvironment. The diagram illustrates four distinct molecular pathways by which exosomes confer therapeutic resistance: (1) HER2^+^ breast cancer exosomes transport TGF-β1 and PD-L1 to induce trastuzumab resistance; (2) Stromal-derived miRNA-21 activates APAF-1 in ovarian cancer cells, leading to paclitaxel resistance; (3) CAF-derived circZFR suppresses the STAT3/NF-κBpathway in hepatoma cells, promoting cisplatin resistance; and (4) Exosomal RN7SL1 triggers RIG-I activation to facilitate therapy resistance. Green arrows indicate activation, while red T-bars indicate inhibition.

The ability of tumor cells to evade immune surveillance has been regarded as one of the key features of cancer metastasis and contributes to the development of drug resistance. Exosomes can directly efflux drugs through various drug transport proteins (Fletcher et al., 2016). Exosomes derived from drug-resistant tumor cells can transfer drug efflux pumps to drug-sensitive cells, causing drug efflux within the receptor cells and thereby reducing the toxicity of the drugs. TDEs can reduce the efficacy of drugs by means of neutralizing antibody-based drugs. In chronic lymphocytic leukemia, exosomal CD20 has been verified to intercept the anti-CD20 antibody rituximab and reduce its efficacy on account of attenuation of the antibody deposition on target cells (Paggetti et al., 2015). Furthermore, HER^2+^ breast cancer cells acquire resistance to trastuzumab by secreting exosomes that contain immunosuppressive cytokines TGFβ1 and the lymphocyte activation inhibitor PD-L1 (Martinez et al., 2017). Recent studies have shown that the contents of exosomes include proteins, RNA and DNA that are involved in tumor immune regulation (Kurywchak et al., 2018). In ovarian cancer, exosomal miRNA-21 released by adipocytes and fibroblasts related to the occurrence of ovarian cancer, activates the miRNA-21/apoptotic protease activating factor-1 (APAF-1) pathway by secreting exosomes, thereby activating the downstream target APAF-1 of miRNA-21, leading to drug resistance of ovarian cancer cells to paclitaxel (Au Yeung et al., 2016). Exosomes derived from tumor-associated fibroblasts can deliver circZFR to hepatoma cells, inhibit the STAT3/NF-κB pathway, and enhance the cisplatin resistance of hepatoma cells (Zhou et al., 2022). TDEs can promote therapy-resistance by carrying unshielded RN7SL1 which activate the pattern recognition receptor (PRR) retinoic acid-inducible gene I (RIG-I) (Nabet et al., 2017) (Figure 7).

### 5.6 Exosomes in drug delivery systems

As a natural “liposome”, exosomes have multiple key attributes, such as biocompatibility, low immunogenicity, low toxic side effects, easy penetration through biological membranes (including the blood-brain barrier), inherent targeting, and can deliver various types of therapeutic drugs, including nucleic acid drugs, protein peptide drugs and small molecule drugs, which have important potential in drug delivery systems (Zhuo et al., 2024). The exosome drug delivery system is composed of exosomes and their contents, that is, loading relevant drugs into exosomes and targeting them to transport to target cells to achieve therapeutic effects. In recent years, exosomes, as a drug delivery system, have demonstrated great potential in the treatment of various diseases such as cancer, mental disorders, central nervous system diseases, and viral diseases (Wang et al., 2023; Meng et al., 2020; Patil et al., 2020; Sarkar et al., 2024). Wang et al. (2024b) constructed a “dual-targeting” anti-cancer drug delivery platform by combining superparamagnetic iron oxide nanoparticles (SPIONs) with exosomes derived from NSCLC cells. It was found that DOX-loaded exosomes-spions (Exo-SPIONs) exhibited optimal tumor tissue delivery and tumor suppression under the action of an external magnetic field, and reduced the toxicity of DOX to normal tissues. Recently, Xiao et al. (2025) discovered that macrophages with chimeric antigen receptors (CARs) generated *in situ* by mRNA delivered by small EVs could alleviate lung cancer metastasis and recurrence. Zhang et al. (De Abreu et al., 2020) coupled cyclo (arginine-glycineaspartate-D tyrosine-lysine)peptide, c (RGDyK) with exosomes derived from bone marrow mesenchymal stem cells, and then loaded cholesterol-modified miRNA-210 onto these exosomes. They found that this exosome could promote angiogenesis in the brain.

The engineered exosome technology utilizes the natural properties of exosomes and uses engineering methods to endow them with additional functional capabilities, providing a more comprehensive strategy for disease diagnosis and treatment (Wang et al., 2025). The study found that engineered RGD-modified Regulatory T cells (Tregs) exosomes (RGD-Treg-Exos) targeted the delivery of miR-218-5p, which activated mitochondrial autophagy and alleviated podocyte damage in diabetic nephropathy (Guo et al., 2025). Fe65-engineered neuronal exosomes carried Corynoxine-B, an autophagy inducer, to the amyloid-β precursor protein (APP) overexpressed-neuron cells mice, which induced autophagy in APP-expressing neuronal cells in the brain of Alzheimer’s disease (AD). It can ameliorate the cognitive decline and pathogenesis in AD mice (Iyaswamy et al., 2023).

## 6 Challenges and future directions

While exosomes exhibit tremendous clinical potential as diagnostic biomarkers and drug delivery vectors, their successful clinical translation is hindered by significant practical barriers. The core challenge remains the lack of standardized protocols, leading to inconsistencies in exosome yield, purity, and biological activity across different laboratories and sample processing times. As drug delivery carriers, engineered EVs enable targeted, efficient drug delivery, though challenges remain in loading efficiency, targeting specificity, and clinical translation.

Adherence to MISEV recommendations is crucial for the standardization of exosome research. Aligned with MISEV 2023 guidelines (Welsh et al., 2024), standardization of isolation, characterization, and reporting is foundational to overcoming these bottlenecks. Regarding isolation, MISEV emphasizes selecting methods tailored to the intended downstream application and acknowledging the inherent limitations of each technique. For characterization, the guidelines mandate the assessment of specific protein markers (including both EV-enriched and non-EV co-isolated proteins) and the use of single-vesicle analysis tools to verify size and morphology. Furthermore, transparent reporting of all experimental parameters is essential to allow for cross-study comparisons and validation. Beyond laboratory standardization, translating exosome research into clinical practice requires overcoming substantial manufacturing and regulatory hurdles. Scalability is a primary bottleneck; transitioning from small-scale benchtop isolations to large-scale, Good Manufacturing Practice (GMP)-compliant production systems (e.g., bioreactors) is required for therapeutic applications. Additionally, inherent biological heterogeneity leads to batch-to-batch variability, necessitating robust QC measures. Rigorous QC must evaluate not only vesicle concentration and purity but also sterility, endotoxin levels, and functional potency. Finally, navigating regulatory considerations—such as defining the active pharmaceutical ingredient (API), assessing pharmacokinetics, and establishing standardized safety profiles under FDA or EMA frameworks—is imperative for the approval of exosome-based therapeutics.

Notably, these challenges are amplified in the context of EV-miRNA-based discoveries. Despite their significant therapeutic and diagnostic outlook, clinical integration is specifically impeded by analytical considerations, including the lack of unified miRNA measurement techniques and the absence of universal endogenous controls for accurate normalization. Addressing these methodological barriers and establishing regulatory structures adapted to the complexity of these nucleotide-carrying biologicals are essential to uphold the rigorous standards of reproducibility and safety necessary for successful clinical translation.

## 7 Conclusion

Cells form a signaling transmission network system among each other through exosomes as a carrier, which mediates interactions between tumor cells or between tumor cells and stromal cells. From inducing the malignant transformation of normal cells, participating in the regulation of TEM, influencing the generation of tumor cell resistance, to regulating the ecological niche of tumor metastasis, it can be proved that exosomes are closely related to tumor occurrence and development. At the same time, exosomes released by tumor cells can also activate immune cells and trigger the immune response of the body. Therefore, exosomes can act as both promoters and inhibitors of tumor development. This provides unlimited possibilities for future therapies aimed at inhibiting tumor growth through researching on suppressing TDEs, drug loading of exosomes, targeted therapies, and many other treatments.

Exosomes can be easily obtained from most body fluids such as blood, urine, saliva and cerebrospinal fluid, and can be used as a tool for liquid biopsy. By detecting the specific biomarkers carried by exosomes, not only can early diagnosis and screening of tumors be achieved, but they can also be used in combination with traditional tumor markers to further enhance diagnostic capabilities, demonstrating extremely high clinical research value in the early diagnosis of tumors. Exosomes have shown a broad application prospect as drug delivery carriers in the treatment of specific diseases. By engineering and modifying exosomes, efficient and targeted delivery of drugs can be achieved. However, fully realizing this potential requires a deeper understanding of the *in vivo* mechanisms governing exosome biogenesis and secretion, which currently remain elusive due to the reliance on *in vitro* models that cannot fully capture individual systemic heterogeneity. Thus, further exploration of EV biogenesis and secretion mechanisms, particularly in in vivo experimental systems, will be critical to elucidating their physiological functions and advancing tumor therapeutic strategies.

Moving forward, bridging the gap between basic research and clinical application will depend on the widespread adoption of consensus guidelines to resolve methodological discrepancies. By surmounting the current bottlenecks in standardization, scalable manufacturing, and rigorous quality control, exosome research is poised to provide a highly favorable theoretical and practical basis for revolutionary tumor treatments.

## Glossary

APAF-1: apoptotic protease activating factor-1
APP: amyloid-β·precursor protein
CAFs: cancer-associated fibroblasts
CARs: chimeric antigen receptors
DEP: dielectrophoresis
DGKa: diacylglycerol kinase
ECM: extracellular matrix
EE: early endosome
EMT: epithelial-mesenchymal transition
ESCRT: endosomal sorting complex required for transport
EVs: extracellular vesicles
HSP: heat shock protein
ILVs: intraluminal vesicles
IncRNA: long non-coding RNA
LBPA: lyosbisphosphatidic acid
LE: late endosome
MVBs: multivesicular bodies
miRNA: microRNA
mRNA: messenger RNA
NSCLC: non-small-cell lung cancer
PBMCs: peripheral blood mononuclear cells
PEG: polyethylene glycol
PLP: proteolipid protein
PKM2: pyruvate kinase type M2
PRR: pattern recognition receptor
RIG-I: retinoic acid-inducible geneI
SNARE: soluble NSF-attachment protein receptor
SEC: size.exclusion chromatography
SPIONs: superparamagnetic iron oxide nanoparticles
TDEs: tumor-derived exosomes
TFF: tangential.flow.filtration
TFR: transferrin receptor
TME: tumor microenvironment
VAMP7: vesicle-associated membrane protein 7
